# Supplementary material for: The impact of the COVID-19 lockdown on birthweight among singleton term birth in Denmark
Source: PLoS One. 2023 Apr 20;18(4):e0283909. doi: 10.1371/journal.pone.0283909 (PMC10118089; doi:10.1371/journal.pone.0283909)
Supplement: Attachment — Submitted filename: Plos One_Response to reviewers_2023_02_10.docx [file pone.0283909.s001.docx]

**Response to Reviewers**

Title: *The impact of the COVID-19 lockdown on birthweight among singleton term birth in Denmark*

Manuscript ID: PONE-D-22-31109

Dear PLOS ONE,

Thank you for considering our manuscript for publication and giving us an opportunity to submit a revised version of out manuscript. You will find our response to journal requirements (point 1-8) and to reviewer #1 (point 1-6) below.

Response to Journal requirements (point 1-8):

1. Please ensure that your manuscript meets PLOS ONE's style requirements, including those for file naming. The PLOS ONE style templates can be found at

<https://journals.plos.org/plosone/s/file?id=wjVg/PLOSOne_formatting_sample_main_body.pdf> and

<https://journals.plos.org/plosone/s/file?id=ba62/PLOSOne_formatting_sample_title_authors_affiliations.pdf>

-> Response: The manuscript now meets PLOS ONES’s style requirements. The manuscript fulfills the advised templates.

2. For studies reporting research involving human participants, PLOS ONE requires authors to confirm that this specific study was reviewed and approved by an institutional review board (ethics committee) before the study began. Please provide the specific name of the ethics committee/IRB that approved your study, or explain why you did not seek approval in this case.

Once you have amended this/these statement(s) in the Methods section of the manuscript, please add the same text to the “Ethics Statement” field of the submission form (via “Edit Submission”).

For additional information about PLOS ONE ethical requirements for human subjects research, please refer to <http://journals.plos.org/plosone/s/submission-guidelines#loc-human-subjects-research>.

-> Response: The study was conducted according to Danish legislation and guidelines for register research stating that anonymized register-based research does not require individual patient consent or ethical approval. This study was accordingly approved be the Data Protection Agency Officer at Statens Serum Institut (No: 20/04753).

3. Please provide additional details regarding participant consent. In the ethics statement in the Methods and online submission information, please ensure that you have specified (1) whether consent was informed and (2) what type you obtained (for instance, written or verbal, and if verbal, how it was documented and witnessed). If your study included minors, state whether you obtained consent from parents or guardians. If the need for consent was waived by the ethics committee, please include this information.

If you are reporting a retrospective study of medical records or archived samples, please ensure that you have discussed whether all data were fully anonymized before you accessed them and/or whether the IRB or ethics committee waived the requirement for informed consent. If patients provided informed written consent to have data from their medical records used in research, please include this information.

Once you have amended this/these statement(s) in the Methods section of the manuscript, please add the same text to the “Ethics Statement” field of the submission form (via “Edit Submission”).

For additional information about PLOS ONE ethical requirements for human subjects research, please refer to <http://journals.plos.org/plosone/s/submission-guidelines#loc-human-subjects-research>.

-> Response: The study was conducted according to Danish legislation and guidelines for register research stating that anonymized register-based research does not require individual patient consent or ethical approval. This study was accordingly approved be the Data Protection Agency Officer at Statens Serum Institut (No: 20/04753).

4. Thank you for stating the following in the Competing Interests section:

“I have read the journal's policy and the authors of this manuscript have the following competing interest: Dr Breindahl has a patent (NeoHelp) with royalties paid. Dr Breindahl has nothing else to disclose.

All other authors reported to have nothing to disclose.”

Please confirm that this does not alter your adherence to all PLOS ONE policies on sharing data and materials, by including the following statement: "This does not alter our adherence to PLOS ONE policies on sharing data and materials.” (as detailed online in our guide for authors <http://journals.plos.org/plosone/s/competing-interests>).  If there are restrictions on sharing of data and/or materials, please state these. Please note that we cannot proceed with consideration of your article until this information has been declared.

Please include your updated Competing Interests statement in your cover letter; we will change the online submission form on your behalf.

-> Response: The statement ‘This does not alter our adherence to PLOS ONE policies on sharing data and materials’ has been added to both the cover letter and the competing interest statement.

5. Thank you for stating the following in the Acknowledgments Section of your manuscript:

“This research was conducted using the Danish Neonatal Screening Biobank and the Danish National Biobank resource, funded by the Novo Nordisk Foundation.”

We note that you have provided additional information within the Acknowledgements Section that is not currently declared in your Funding Statement. Please note that funding information should not appear in the Acknowledgments section or other areas of your manuscript. We will only publish funding information present in the Funding Statement section of the online submission form.

Please remove any funding-related text from the manuscript and let us know how you would like to update your Funding Statement. Currently, your Funding Statement reads as follows:

“The author(s) received no specific funding for this work.”

Please include your amended statements within your cover letter; we will change the online submission form on your behalf.

-> Response: The funding information has been removed from the acknowledgements. We do not wish to add anything to our funding statement.

6. In your Data Availability statement, you have not specified where the minimal data set underlying the results described in your manuscript can be found. PLOS defines a study's minimal data set as the underlying data used to reach the conclusions drawn in the manuscript and any additional data required to replicate the reported study findings in their entirety. All PLOS journals require that the minimal data set be made fully available. For more information about our data policy, please see <http://journals.plos.org/plosone/s/data-availability>.

Upon re-submitting your revised manuscript, please upload your study’s minimal underlying data set as either Supporting Information files or to a stable, public repository and include the relevant URLs, DOIs, or accession numbers within your revised cover letter. For a list of acceptable repositories, please see <http://journals.plos.org/plosone/s/data-availability#loc-recommended-repositories>. Any potentially identifying patient information must be fully anonymized.

Important: If there are ethical or legal restrictions to sharing your data publicly, please explain these restrictions in detail. Please see our guidelines for more information on what we consider unacceptable restrictions to publicly sharing data: <http://journals.plos.org/plosone/s/data-availability#loc-unacceptable-data-access-restrictions>. Note that it is not acceptable for the authors to be the sole named individuals responsible for ensuring data access.

We will update your Data Availability statement to reflect the information you provide in your cover letter.

-> Response: Relevant data are within the manuscript. Full data cannot be shared publicly due to data protection regulations. Data are available from Statens Serum Institut (contact via corresponding author MC, [mic@ssi.dk](mailto:mic@ssi.dk)) for researchers who meet the criteria for access to confidential data.

7. Your ethics statement should only appear in the Methods section of your manuscript. If your ethics statement is written in any section besides the Methods, please delete it from any other section.

-> Response: Our ethics statement now only appears in the Methods section of the manuscript. The ethics statement that appeared on the title page has now been removed.

8. Please review your reference list to ensure that it is complete and correct. If you have cited papers that have been retracted, please include the rationale for doing so in the manuscript text, or remove these references and replace them with relevant current references. Any changes to the reference list should be mentioned in the rebuttal letter that accompanies your revised manuscript. If you need to cite a retracted article, indicate the article’s retracted status in the References list and also include a citation and full reference for the retraction notice.

-> Response: We have reviewed our reference list and have made no changes.

Reponse to Reviewer #1 (point 1-6):

1. Is the manuscript technically sound, and do the data support the conclusions?

The manuscript must describe a technically sound piece of scientific research with data that supports the conclusions. Experiments must have been conducted rigorously, with appropriate controls, replication, and sample sizes. The conclusions must be drawn appropriately based on the data presented.

Reviewer #1: Yes

-> Response: Thank you.

2. Has the statistical analysis been performed appropriately and rigorously?

Reviewer #1: Yes

-> Response: Thank you.

3. Have the authors made all data underlying the findings in their manuscript fully available?

The [PLOS Data policy](http://www.plosone.org/static/policies.action#sharing) requires authors to make all data underlying the findings described in their manuscript fully available without restriction, with rare exception (please refer to the Data Availability Statement in the manuscript PDF file). The data should be provided as part of the manuscript or its supporting information, or deposited to a public repository. For example, in addition to summary statistics, the data points behind means, medians and variance measures should be available. If there are restrictions on publicly sharing data—e.g. participant privacy or use of data from a third party—those must be specified.

Reviewer #1: Yes

-> Response: we agree. Specifically, we have specified our data sharing policy and reason herefor above and in the manuscript.

4. Is the manuscript presented in an intelligible fashion and written in standard English?

PLOS ONE does not copyedit accepted manuscripts, so the language in submitted articles must be clear, correct, and unambiguous. Any typographical or grammatical errors should be corrected at revision, so please note any specific errors here.

Reviewer #1: Yes

-> Response: Thank you.

5. Review Comments to the Author

Please use the space provided to explain your answers to the questions above. You may also include additional comments for the author, including concerns about dual publication, research ethics, or publication ethics. (Please upload your review as an attachment if it exceeds 20,000 characters)

Reviewer #1: I've read with interest the paper by De Knegt et al. about the impact of the COVID-19 lockdown on birthweight among singleton term birth in Denmark. The study follows the scientific rigor and the manuscript is well written.
I have only one comment, Would the results be the same if the authors use local birthweight charts?

-> Response: We thank the reviewer for their question. In this study, we defined SGA as birth weight less than the 10^th^ percentile and LGA greater than the 90^th^ percentile at each completed week, for each sex, according to the international standards of newborn size from the INTERGROWTH-21^st^ Project. Data from the INTERGROWTH 21^st^ Project stem from eight different urban populations, and allows for comparisons across multiethnic populations.

To ensure accuracy and appropriateness within our population, we also defined SGA and LGA as births with birth weight, corrected for sex and gestational week, below the 5^th^ percentile or above the 95^th^ percentile of the study population from 2015 - 2019, respectively. This thereby serves as an internal verification using local, Danish data.

We decided against using e.g. the Scandinavian intrauterine growth curves developed by Marsal et. Al. in 1996. These growth curves are intrauterine curves based on ultrasound-estimated foetal weights in women in Denmark and Sweden. We do not feel that these growth curves constitute appropriate local growth curves for birth weight. Back in 1996, ca. 24% of Danish and Swedish women were smokers. Today between 5-8% of pregnant women smoke. Furthermore, pregnant women living in Denmark today are more ethnically heterogenous than 26-years ago. Finally, the curves created by Marsal et al. were intended to understand intrauterine growth, not birth weight. No other appropriate local growth curves exist, and as such, the curves from the INTERGROWTH 21^st^ Project as well as our own internal reference for LGA and SGA seem most appropriate.

Regarding the wish of a reference to local weight charts, we believe we have met this as we refer to 95-percentile values from our own population - after having established that there was no significant change from 2015-2019 (table 3). Our findings regarding an increase in the proportion of LGA infants was apparent using both the INTERGROWTH 21 curves and our own internal cut-off percentiles from our own population.

To clarify this, we have added a sentence to the manuscript to address the concern of not using a local/Danish growth curves: Line 281-284: ‘Furthermore, our approach of validating our findings using the international reference from the INTERGROWTH 21^st^ Project by also referring to 95-percentile values for SGA and LGA in our own population addresses any issues concerning the lack of use of more local/Danish growth curves.’

6. PLOS authors have the option to publish the peer review history of their article ([what does this mean?](https://journals.plos.org/plosone/s/editorial-and-peer-review-process#loc-peer-review-history)). If published, this will include your full peer review and any attached files.

If you choose “no”, your identity will remain anonymous but your review may still be made public.

**Do you want your identity to be public for this peer review?** For information about this choice, including consent withdrawal, please see our [Privacy Policy](https://www.plos.org/privacy-policy).

Reviewer #1: No
